# Mucosal and systemic immune signatures reveal compartmentalized regulation of gut barrier integrity in virologically suppressed HIV Infection

**DOI:** 10.3389/fimmu.2026.1876176

**Published:** 2026-07-15

**Authors:** Valentina Artusa, Roberta Zamarato, Lara De Luca, Livia Benedetti, Mirko Compagno, Mario Alberto Cano Fiestas, Diego Bottan, Mario Clerici, Mara Biasin, Daria Trabattoni, the MARISA Study Group

**Affiliations:** 1 Department of Biomedical and Clinical Sciences, University of Milan, Milan, Italy; 2 Department of System Medicine, Tor Vergata University, Rome, Italy; 3 Department of Clinical Sciences and Community Health, University of Milan, Milan, Italy; 4 Department of Pathophysiology and Transplantation, University of Milan, Milan, Italy; 5 Istituto di Ricovero e Cura a Carattere Scientifico (IRCCS) Fondazione Don Carlo Gnocchi ONLUS, Milan, Italy

**Keywords:** HIV infection, gut mucosal immunity, clinical disease stage, integrase strand transfer inhibitor, immune recovery

## Abstract

Persistent immune activation and inflammation contribute to non–AIDS comorbidities in people living with HIV (PLWH) despite long-term virological suppression. Given the central role of the gut in immune homeostasis, we investigated whether mucosal and peripheral molecular signatures relate to clinical status, immune recovery, and antiretroviral therapy (ART) class. Sixty-eight virologically suppressed PLWH were stratified by CDC clinical stage, ART regimen (integrase strand transfer inhibitor [INSTI]–based vs. non-INSTI), and CD4/CD8 ratio. Targeted gene expression was assessed by RT-PCR in anorectal mucosal biopsies and peripheral blood mononuclear cells, while epithelial protein expression was evaluated by Western blot in mucosal biopsies. Plasma biomarkers of epithelial injury, microbial translocation, and systemic inflammation were quantified by ELISA. Group comparisons and correlation analyses were performed using non-parametric statistics. Mucosal analyses revealed distinct transcriptional and protein-level alterations associated with clinical and immunological stratifications, whereas peripheral cellular and circulating biomarkers showed limited discriminatory capacity. Notably, specific ART-related patterns emerged at the mucosal level, accompanied by coordinated associations between epithelial junctional features and local immune parameters that were not mirrored systemically. Together, these findings indicate persistent, spatially restricted epithelial–immune remodelling in treated HIV infection, underscoring the importance of direct mucosal assessment to capture residual barrier and immune perturbations under suppressive ART.

## Highlights

- Distinct mucosal transcriptional patterns reflect disease stage, ART regimen and immune recovery.
- INSTI-based therapy drives epithelial junctional remodelling toward improved barrier integrity.
- Analyses reveal coordinated but spatially restricted epithelial–immune regulation in treated HIV.

## 1 Introduction

The intestinal barrier represents a multifaceted anatomical and functional interface between the host immune system and the vast luminal content of the gastrointestinal tract, including dietary antigens, commensal microbiota, and potential pathogens. This barrier is composed of a single layer of intestinal epithelial cells (IECs) connected by apical junctional complexes, tight junctions (TJs), adherens junctions, and desmosomes, overlain by a protective mucus layer enriched with antimicrobial peptides (AMPs) and secretory immunoglobulin A (sIgA) (1, 2). Beneath the epithelium lies the lamina propria, a compartment densely populated by immune cells, including T and B lymphocytes, dendritic cells, and macrophages, which orchestrate a finely tuned balance between immune tolerance and active defence (3, 4).

In the setting of human immunodeficiency virus (HIV) infection, this intricate system undergoes profound and sustained perturbation (5, 6). One of the earliest and most striking immunopathological events following mucosal HIV exposure is the rapid and preferential depletion of CCR5^+^ CD4^+^ T cells in gut-associated lymphoid tissue (GALT), including Th17 and Th22 subsets. These subsets are critical for the maintenance of epithelial integrity, regulation of antimicrobial peptide expression, and promotion of mucosal barrier function. Their loss not only impairs local immune responses but also facilitates a breakdown in epithelial homeostasis (7).

Concomitant to this immune depletion, HIV exerts direct cytopathic effects on IECs and disrupts epithelial junctional complexes, contributing to increased paracellular permeability. Inflammatory cytokines such as TNF, IFN-γ, and IL-1β, upregulated in response to both viral replication and microbial stimuli, further destabilize TJ architecture by modulating the expression and phosphorylation status of key proteins such as claudins, occludin, and zonula occludens-1 (ZO-1). This cascade of events culminates in the translocation of microbial products, including lipopolysaccharide (LPS), flagellin, peptidoglycan, and bacterial DNA, from the intestinal lumen into systemic circulation, a process termed microbial translocation.

Microbial translocation is a potent driver of chronic immune activation, which in turn promotes further CD4^+^ T cell depletion and contributes to lymphoid tissue fibrosis. These processes create a self-perpetuating cycle of mucosal damage, inflammation, and immune dysfunction. Notably, this pathological axis persists despite the initiation of suppressive combination antiretroviral therapy (cART). Although cART effectively suppresses plasma viraemia and partially restores peripheral CD4^+^ T cell counts, it does not fully reconstitute mucosal immunity nor reverse the structural and functional defects of the intestinal barrier. Evidence from clinical and translational studies (8–11), indicates that ART-treated individuals continue to exhibit elevated levels of circulating microbial products, systemic inflammatory biomarkers (e.g., soluble CD14, IL-6, LBP), and markers of epithelial turnover (e.g., intestinal fatty acid–binding protein, I-FABP), suggestive of ongoing barrier dysfunction.

Furthermore, several factors may limit mucosal healing during ART, including residual viral replication in tissue reservoirs, persistent dysbiosis of the gut microbiota, impaired IL-22 signalling, and fibrotic remodelling of the lymphoid architecture. The incomplete resolution of intestinal barrier dysfunction has significant clinical implications, as it has been independently associated with the development of non-AIDS comorbidities, including cardiovascular disease (12, 13), insulin resistance (14, 15), liver fibrosis (16, 17), and neurocognitive impairment (18, 19), conditions now recognized as major contributors to morbidity and mortality in the ART era.

Considering these findings, the gut has emerged as a central organ in HIV pathogenesis, not merely as a site of early immune depletion but as a long-term contributor to systemic disease (20). A deeper understanding of the mechanisms underlying intestinal barrier disruption and its persistence despite effective viral suppression is crucial. Such insights are essential for guiding the development of novel therapeutic interventions aimed at mucosal repair, restoration of barrier integrity, and attenuation of systemic inflammation in people living with HIV (PLWH).

Despite extensive research, several critical gaps remain in our understanding of intestinal barrier dysfunction in the context of chronic, virologically suppressed HIV infection. Most studies have examined individual compartments, mucosal, peripheral, or systemic, in isolation, limiting the ability to define how local epithelial perturbations translate into systemic immune activation. Furthermore, the molecular underpinnings linking epithelial gene regulation, immune signalling, and circulating biomarkers remain poorly characterized, particularly in human mucosal tissue. The potential modulatory effects of distinct ART classes, including integrase strand transfer inhibitor (INSTI)–based regimens, on epithelial junctional integrity and mucosal immune homeostasis have yet to be systematically investigated. Finally, whether immunological recovery, as reflected by the CD4^+^/CD8^+^ ratio or CDC clinical stage, parallels restoration of epithelial barrier function remains unclear. Addressing these knowledge gaps requires an integrated, multi-compartmental analysis that concurrently evaluates mucosal, peripheral, and systemic markers of barrier integrity and immune activation. The present study was therefore designed to comprehensively assess these interrelated processes in PLWH under suppressive ART, providing new insights into the persistence and compartmentalization of epithelial-immune dysregulation in the context of long-term treated HIV infection.

## 2 Materials and methods

### 2.1 Enrolment and clinical evaluation

Beginning in July 2022, 85 virologically suppressed PLWH were enrolled in the MARISA study (Project No. 202074AA2B), an observational project investigating viro-immunological and mucosal parameters in individuals remaining on stable 3-drug ART regimens. or switching from 3-drug to 2-drug ART. As this study was based on baseline sampling, all individuals were receiving standard 3-drug antiretroviral therapy at the time of analysis. Peripheral blood and anorectal mucosal biopsies (via high-resolution anoscopy) were collected to assess immune, virological, and pharmacological markers in blood and GALT.

At enrolment, clinical and demographic data (including sex, age, CD4^+^ nadir, HIV RNA zenith, current CD4^+^/CD8^+^ counts, plasma HIV RNA, ART regimen, ART initiation date, and AIDS history) were recorded for participant stratification and correlation with gut barrier dysfunction and systemic immune activation markers. All study procedures were conducted in accordance with the Declaration of Helsinki and approved by the Institutional Ethics Committee (Protocol No. 111/19), with written informed consent obtained from all participants.

### 2.2 Participants stratification

Three predefined clinical and immunological parameters were used to define comparison groups, allowing a comprehensive assessment of factors potentially influencing intestinal barrier integrity and immune activation.

Participants were first classified according to the Centers for Disease Control and Prevention (CDC) clinical staging system (stages A, B, and C) (21). This stratification reflects the extent of immunological and clinical deterioration, allowing assessment of whether progressive disease stage is associated with increased intestinal permeability and microbial translocation. Previous studies have demonstrated that advanced clinical stages correlate with heightened systemic immune activation and microbial translocation, implicating mucosal damage as a key contributor to HIV pathogenesis (22).

PLWH enrolled in the study were also stratified according to their ART regimen, specifically by according to whether they were using INSTIs or not. INSTI-based regimens represent the current standard of care due to their potent antiviral efficacy, rapid viral suppression, and favourable tolerability profile (23). However, emerging evidence indicates that INSTIs may differentially influence immune activation, gut microbiota composition, and inflammatory pathways compared with other ART classes (24, 25). Stratifying by regimen therefore allowed the investigation of whether INSTI exposure modulates epithelial barrier integrity and mucosal immune homeostasis.

A third subgrouping variable was the CD4^+^/CD8^+^ T-cell ratio, distinguishing participants with immune dysregulation (ratio <1) from those with more complete immune reconstitution (ratio ≥1) (26). This parameter serves as an established marker of long-term immune competence in PLWH. A CD4^+^/CD8^+^ ratio <1 has been associated with persistent immune activation, enhanced microbial translocation, and an increased risk of non-AIDS comorbidities, even in individuals with suppressed plasma viraemia. Incorporating this measure enabled a refined analysis of immune recovery in relation to intestinal barrier function beyond absolute CD4^+^ T-cell counts.

Together, these three complementary stratification criteria (clinical stage, ART regimen, and CD4^+^/CD8^+^ ratio) provided a structured framework for examining demographic, virological, and immunological characteristics in relation to molecular and biochemical markers of intestinal barrier function.

### 2.3 Plasma and PBMCs collection

Whole blood was collected in EDTA-coated BD Vacutainer^®^ K2E tubes and centrifuged at 1,500 × g for 10 minutes at room temperature to separate plasma, which was carefully aspirated, aliquoted, and stored at –80 °C. The remaining blood was diluted 1:2 with PBS (pH 7.4), in Leucosep™ tubes (50 mL; Greiner Bio-One). Samples were centrifuged at 800 × g, for 30 minutes without brake. PBMCs were collected from the interphase, washed with PBS. No red blood cell lysis was performed, as erythrocyte separation was achieved by the integrated porous barrier of the Leucosep™ tubes. After a final wash, cells were resuspended in cryopreservation medium (90% foetal bovine serum (FBS), 10% dimethyl sulfoxide), aliquoted, and cryopreserved in liquid nitrogen. Cryopreserved samples were shipped in batches on dry ice, with shipment duration not exceeding 24 hours.

### 2.4 Anorectal biopsies collection

Rectal mucosal biopsies were collected from PLWH during routine screening for genito-anorectal HPV infection and anal cancer, following informed consent and ethical guidelines. High-resolution anoscopy (HRA) was performed using the THD^®^ Procto-Station and HR Light Scope Procto. Four to eight biopsies per participant were obtained with 2.45 mm sterile forceps from macroscopically normal mucosa located 10–20 cm from the anal verge. Samples were snap-frozen without fixatives. Cryopreserved samples were shipped in batches on dry ice, with a maximum shipping duration of 24 hours.

### 2.5 RNA extraction from RNA STAT-60-homogenized samples

Cryopreserved PBMCs were thawed at 37 °C and transferred to pre-warmed RPMI 1640 medium (Euroclone S.p.A.) with 20% FBS, then incubated overnight at 37 °C with 5% CO_2_. Cells were harvested and lysed in RNA STAT-60 (TEL-TEST, Inc., 200 μL per 1 × 10^6^ cells) for RNA extraction. Dry-frozen anorectal biopsies (0.1–17.5 mg) were homogenized in 600 μL RNA STAT-60. Total RNA was extracted using 1-Bromo-3-chloropropane (BCP, Sigma-Aldrich, 1:5 ratio), followed by centrifugation at 12,000 g for 15 min at 4 °C. The aqueous phase was collected, mixed with isopropanol (1:1), and incubated overnight at –20 °C. RNA was pelleted by centrifugation, washed twice with 75% ethanol, air-dried, and resuspended in 11 μL nuclease-free water. RNA yield and purity were assessed by NanoDrop 2000.

### 2.6 Reverse transcription

One microgram of total RNA per sample was DNase-treated (New England Biolabs) to remove genomic DNA. cDNA was synthesized using Promega reverse transcriptase, oligo(dT), and random primers, following standard protocols, and stored at –20 °C.

### 2.7 Gene expression analysis via real time-PCR

Real-time PCR was performed using 25 ng of cDNA per sample to assess expression of 32 genes (**Supplementary Table S1**). Structural and immunological genes were analysed in anorectal biopsies, while only immunological genes were assessed in PBMCs. Beside GAPDH, and β-actin, RPLP0 and RPS9 were used for normalization in biopsies, based on their stable expression across individuals, inflammatory states, and intestinal regions (27).

### 2.8 Protein purification from RNA STAT-60-homogenized biopsies

Proteins were isolated from RNA STAT-60–homogenized rectal biopsies using a protocol adapted from Wen et al. (28) (detailed in **Supplementary Material**). After RNA extraction, genomic DNA was precipitated with ethanol, and proteins were recovered from the remaining organic phase through sequential precipitation with ethanol, BCP, and distilled water. Protein pellets were washed, air-dried, and resuspended in SDS-urea complete buffer (85% SDS-urea, 12% glycerol, 2% β-mercaptoethanol, 1% protease inhibitors). Samples were incubated at 50 °C for 1 hour, denatured at 95 °C for 5 minutes, and stored at –20 °C until analysis.

### 2.9 Protein quantification

Proteins resuspended in SDS-urea buffer containing detergents and reducing agents were quantified using the RC DC™ Protein Assay Kit I (Bio-Rad), suitable for such conditions, following the manufacturer’s instructions.

### 2.10 Gel electrophoresis and Western blot

For each sample, 30 μg of total protein were separated by SDS-PAGE on 10% Mini-PROTEAN^®^ TGX™ Stain-Free™ gels (Bio-Rad) using 1X Tris/Glycine/SDS Running Buffer. A pre-stained molecular weight ladder (Euroclone) was included as reference. Electrophoresis was run at 100 V for ~90 min, and gels were imaged on a ChemiDoc™ XRS+ system (Bio-Rad) before transfer to PVDF membranes using the Trans-Blot^®^ Turbo™ system. Membranes were blocked with 5% BSA in TBST for 1 h, incubated overnight at 4 °C with primary antibodies against E-cadherin (#14472S) and occludin (#91131S) (1:1000, Cell Signaling Technology), then with HRP-conjugated anti-rabbit secondary antibody (#7074S) for 1 h at room temperature. Detection was performed using Clarity™ Western ECL substrate (Bio-Rad), and signals were acquired and quantified with Image Lab Software (Bio-Rad). Protein expression was normalized to total protein content visualized by stain-free imaging.

### 2.11 ELISA

Plasma samples were thawed on ice and centrifuged to remove particulates. Biomarker quantification was performed using ELISA kits (Assay Genie), according to manufacturer instructions. The following analytes were measured: LPS (UNFI0091), LBP (HUFI01277), sCD14 (HUFI02837), IL-6 (HUFI00180), I-FABP (HUFI02570), REG3α (HUFI01006), TFF3 (HUFI00243), occludin (HUFI01385), zonulin (HUES03536), and E-cadherin (HUFI00102).

### 2.12 Statistical analysis

Statistical analyses were performed using GraphPad Prism v10.2 (GraphPad Software, San Diego, CA) or IBM SPSS Statistics v29.0 (IBM Corp.) software. Gene expression was analysed using normalized Ct values and the ΔΔCt method, with results expressed as fold changes relative to a calibrator (B01, C01). ELISA data were reported as absolute concentrations (pg/mL or ng/mL) based on standard curves.

Descriptive statistics and graphical outputs were used to assess data distribution. Group comparisons were conducted using the Kruskal-Wallis test (CDC stages A vs. B vs. C) or Mann–Whitney U test (INSTI vs. non-INSTI regimens, CD4/CD8 <1 vs. CD4/CD8 >1). To assess the associations between group stratifications and molecular analysis outcomes, we employed linear regression models. Dependent variables were subjected to a natural logarithmic transformation prior to modelling to satisfy the assumption of normality. The regression models were adjusted for relevant covariates, to isolate the independent effect of the group stratifications. Spearman’s rank correlation was employed to evaluate potential associations among all parameters evaluated in the study, including molecular, immunological, and clinical variables, to identify any interrelationships within and across biological compartments. All tests were two-tailed; *p* < 0.05 was considered statistically significant.

## 3 Results

### 3.1 Clinical characteristics of the cohort

Clinical and demographic characteristics of the study participants, including key immunological and virological parameters are summarized in **Table 1**. Although 85 patients were initially recruited, not all samples were suitable for downstream analyses following initial processing; therefore, 68 samples were included in the final molecular and statistical analyses.

**Table 1:** Clinical, demographic, immunological, and virological characteristics of study participants.

Variable	
N=68	
Sex (%M, %MtF, %F)	91.2, 4.4, 4.4
Age (years)	44.6 (± 9.7)
Current CD4^+^ count (cells/mm3)	794 (666-953)
Current CD8^+^ count (cells/mm3)	787 (596-1210)
CD4^+^ nadir (cells/mm3)	385 (± 216)
HIV RNA zenith (copies/mL)	75089 (24881-3300113)
Current HIV RNA (copies/mL)	0 (0-0)
Years of therapy	4 (3-8)

Values are expressed as percentage for sex, as mean (± standard deviation) for normally distributed variables, as median (interquartile range) for non-normally distributed variables. Current plasma HIV RNA values were obtained from routine clinical testing. Values reported as target not detected (TND) or below the assay detection threshold (<20 copies/mL) were assigned an arbitrary value of 0 for statistical analyses.

To enable a more detailed characterization of the study population, participants were stratified according to key clinical and immunological parameters. **Tables 2**–**4** present the resulting group distributions: **Table 2** details participants categorized by clinical stage, **Table 3** compares individuals receiving integrase inhibitor–based versus non–integrase inhibitor–based antiretroviral regimens, and **Table 4** summarizes groups defined by CD4/CD8 ratio strata. Notably, stratification by CDC classification was available for 66 individuals due to missing information for two cases (**Table 2**). This stratification provides a structured framework for evaluating demographic, virological, and immunological characteristics across clinically relevant subgroups.

**Table 2:** Clinical, immunological, and virological characteristics of participants stratified by CDC clinical stage.

Variable		CDC stage		
	A (N = 42)	B (N = 16)	C (N = 6)	*P-value*
Sex (%M, %MtF, %F)	95.2, 2.4, 2.4	81.3, 12.5, 6.3	87.5, 0, 12.5	0.51
Age (years)	42.1 (± 8.6)	46 (± 9.1)	50.2 (± 10.4)	0.09
Current CD4^+^ count (cells/mm3)	834 (702-974)	847 (654-1045)	695 (488-791)	0.07
Current CD8^+^ count (cells/mm3)	706 (551-1059)	848 (646-1234)	1211 (725-1657)	0.08
CD4^+^ nadir (cells/mm3)	477 (± 175)	288 (± 207)*	122 (± 84)**	<0.001
HIV RNA zenith (copies/mL)	59219 (20579- 162000)	91600 (24528- 354853)	551000 (156750- 751250)*	0.01
Current HIV RNA (copies/mL)	0 (0-0)	0 (0-32)	0 (0-18)	0.09

Values are expressed as percentage for sex, as mean (± standard deviation) for normally distributed variables, as median (interquartile range) for non-normally distributed variables. Statistically significant differences vs. Group A are indicated by one or more asterisks (*p < 0.05, **p < 0.01). Current plasma HIV RNA values were obtained from routine clinical testing. Values reported as target not detected (TND) or below the assay detection threshold (<20 copies/mL) were assigned an arbitrary value of 0 for statistical analyses.

**Table 3:** Clinical, immunological, and virological characteristics of participants stratified by antiretroviral regimen (INSTI-based vs. non–INSTI-based therapy).

Variable	Non-INSTI (N = 20)	INSTI (N = 48)	*P-value*
Sex (%M, %MtF, %F)	85, 5, 10	93.8, 4.2, 2.1	0.34
Age (years)	45.2 (± 10.7)	44.3 (± 9.3)	0.55
Current CD4^+^ count (cells/mm3)	830 (680-1151)	793 (651-883)	0.15
Current CD8^+^ count (cells/mm3)	877 (566-1331)	736 (598-1189)	0.43
CD4^+^ nadir (cells/mm3)	410 (± 205)	375 (± 221)	0.60
HIV RNA zenith (copies/mL)	63389 (19852-196469)	90200 (29079-404187)	0.29
Current HIV RNA (copies/mL)	0 (0-22)	0 (0-0)	1.00

Values are expressed as percentage for sex, as mean (± standard deviation) for normally distributed variables, as median (interquartile range) for non-normally distributed variables. Current plasma HIV RNA values were obtained from routine clinical testing. Values reported as target not detected (TND) or below the assay detection threshold (<20 copies/mL) were assigned an arbitrary value of 0 for statistical analyses.

**Table 4:** Clinical, immunological, and virological characteristics of participants stratified by CD4/CD8 ratio category.

Variable	CD4/CD8 < 1 (N = 33)	CD4/CD8 > 1 (N = 35)	*P-value*
Sex (M, MtF, F)	90.9, 6.1, 3	91.4, 2.9, 5.7	0.71
Age (years)	46.8 (± 8.8)	42.4 (± 10.1)*	0.04
Current CD4^+^ count (cells/mm3)	764 (561-890)	846 (711-958)*	0.05
Current CD8^+^ count (cells/mm3)	1208 (848-1542)	601 (502-763)**	<0.01
CD4^+^ nadir (cells/mm3)	316 (± 220)	442 (± 197)*	0.02
HIV RNA zenith (copies/mL)	79336 (48650-359009)	75089 (19345-19345-324938)	0.44
Current HIV RNA (copies/mL)	0 (0-0)	0 (0-0)	0.60

Values are expressed as percentage for sex, as mean (± standard deviation) for normally distributed variables, as median (interquartile range) for non-normally distributed variables. Statistically significant differences between groups are indicated by one or more asterisks (*p < 0.05, **p < 0.01). Current plasma HIV RNA values were obtained from routine clinical testing. Values reported as target not detected (TND) or below the assay detection threshold (<20 copies/mL) were assigned an arbitrary value of 0 for statistical analyses.

As reported in **Table 2**, subjects in stages B and C showed reduced CD4^+^ nadir levels, while stage C results in a higher HIV RNA zenith than stage A. Similarly, **Table 4** shows significant differences in age, current CD4^+^ and CD8^+^ counts, and CD4^+^ nadir depending on the CD4/CD8 ratio. Subjects with a CD4/CD8 ratio greater than 1 were younger and had a higher CD4^+^ and a lower CD8^+^ current count, and a higher CD4^+^ nadir.

### 3.2 Gene expression profiling of rectal biopsies

**Figure 1:**
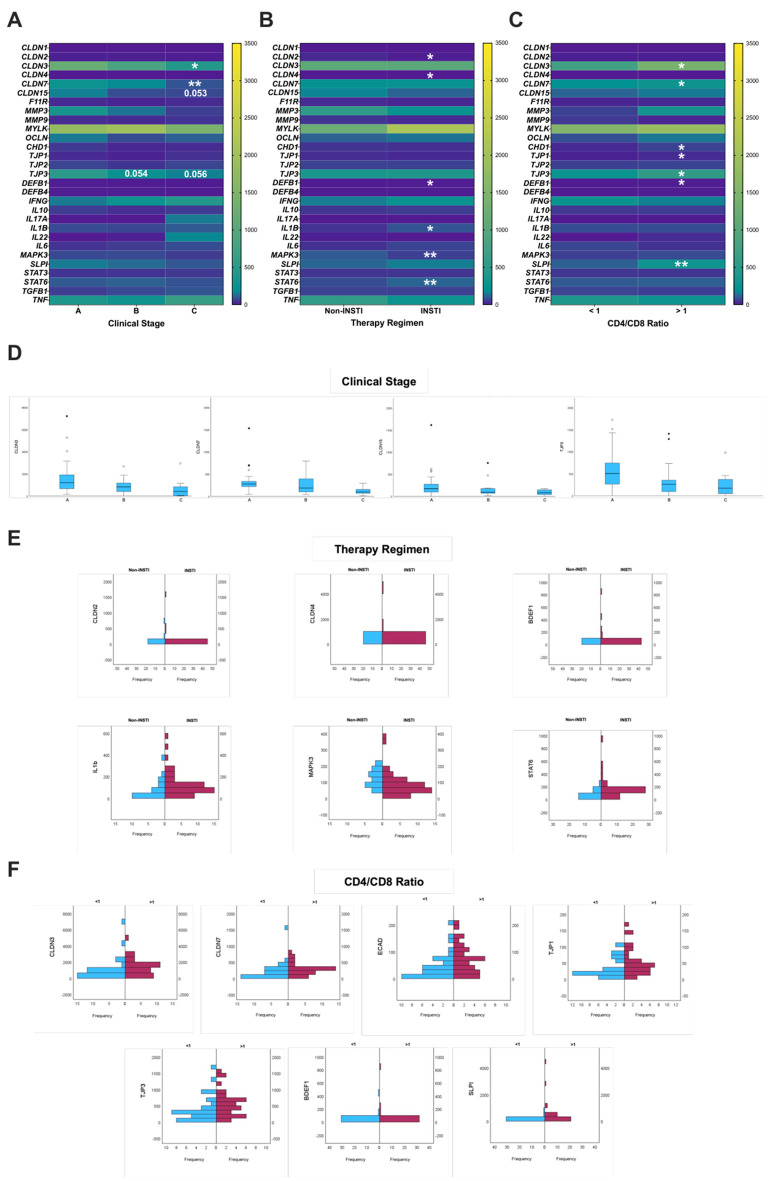
Differential expression of epithelial barrier- and immune-related genes in anorectal mucosa according to clinical and immunological parameters of HIV infection. **(A–C)** Heatmaps summarizing the relative gene expression profiles across participants stratified by **(A)** clinical stage, **(B)** antiretroviral regimen (integrase inhibitor–based *versus* non–integrase inhibitor–based therapy), and **(C)** CD4/CD8 ratio category (>1 *versus* < 1). Genes related to epithelial junctional integrity and innate immune defence are shown. Statistically significant differences between groups are indicated by one or more asterisks (*p < 0.05, **p < 0.01). **(D–F)** Box plots illustrating in detail the expression differences for genes that were significantly modulated across **(D)** clinical stages, **(E)** antiretroviral regimen categories, and **(F)** CD4/CD8 ratio groups. Only transcripts showing statistically significant differential expression are presented.

First, we assessed the expression of epithelial barrier- and immune-related genes in anorectal mucosal biopsies from our cohort. We observed distinct transcriptional patterns of these genes across groups. Individuals at advanced clinical stages (**Figure 1A**) showed reduced expression of *CLDN3*, *CLDN7*, *CLDN15*, and *TJP3*. Participants receiving integrase inhibitor–based regimens (**Figure 1B**) exhibited *CLDN2* downregulation and *CLDN4* upregulation, together with increased expression of *DEFB1*, *IL1B*, and *STAT6*, and decreased *MAPK3* levels. Individuals with a CD4/CD8 ratio >1 (**Figure 1C**) displayed higher expression of *CLDN3*, *CLDN7*, *TJP1*, *TJP3*, *CHD1*, *DEFB1*, and *SLPI* compared with those with lower ratios.

### 3.3 Protein expression in rectal biopsies

**Figure 2:**
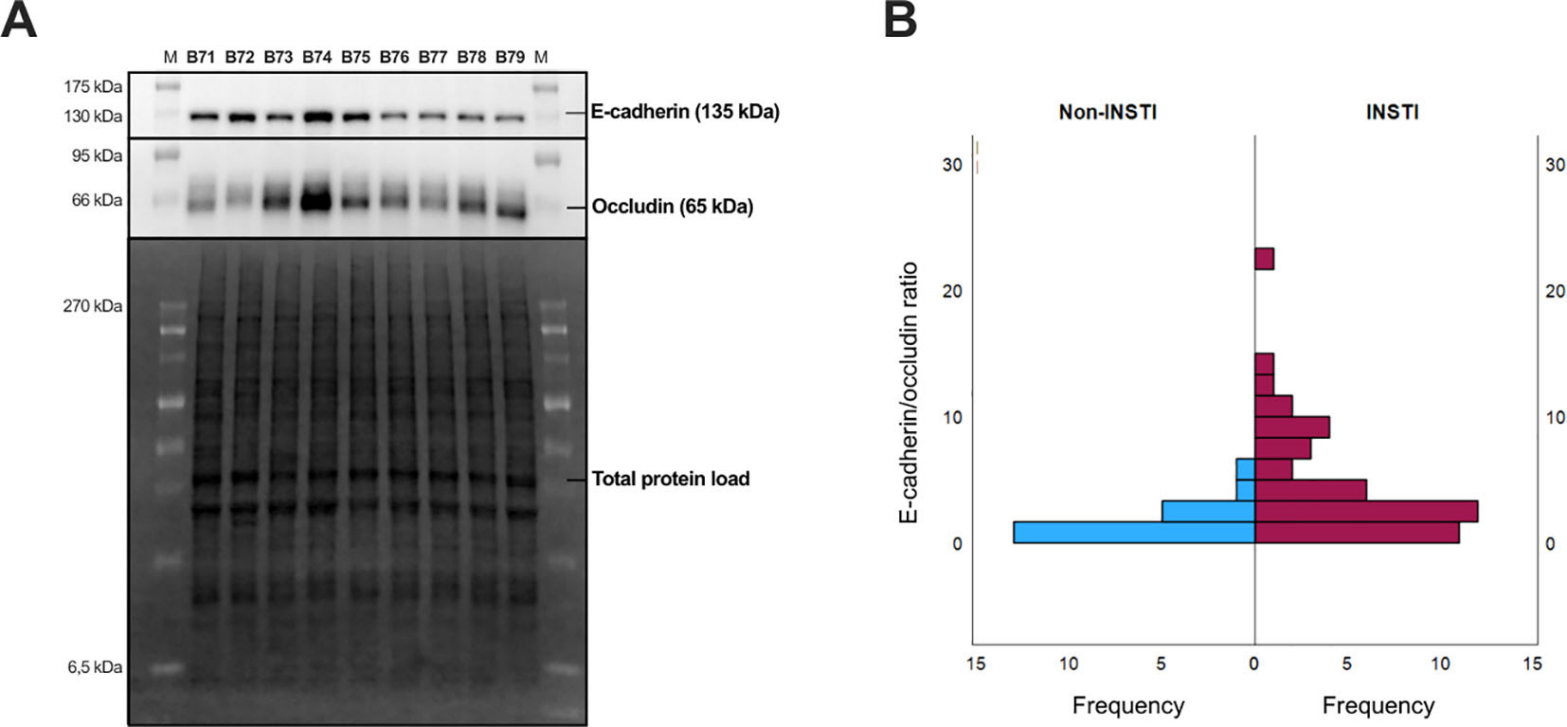
Protein expression of epithelial junctional components E-cadherin and occludin in anorectal mucosal samples. **(A)** Representative immunoblot showing reactive bands for E-cadherin and occludin, together with the total protein load used for normalization. Quantification of band intensity was performed relative to total protein signal to ensure accurate normalization across samples. **(B)** Frequency plots showing the distribution of densitometric values for the E-cadherin/occludin ratio in participants receiving integrase inhibitor–based and non–integrase inhibitor–based antiretroviral regimens (*p* < 0.05).

We next evaluated the protein expression of two key structural components of the epithelial barrier, E-cadherin and occludin, in the same anorectal mucosal samples used for the transcriptomic analysis. This was made possible by an optimized extraction protocol that allowed for the simultaneous isolation of RNA and proteins from the same portion of frozen tissue, thereby ensuring direct comparability between gene and protein expression data. At the individual protein level, no significant differences in E-cadherin or occludin abundance were detected across the three stratifications (clinical stage, antiretroviral regimen, and CD4/CD8 ratio groups) (**Supplementary Figure S2**). However, the E-cadherin/occludin ratio was significantly higher in participants receiving integrase inhibitor–based therapy, suggesting a relative preservation of adherens junction components within this treatment group (**Figure 2B**).

### 3.4 Gene expression profiling of PBMCs

**Figure 3:**
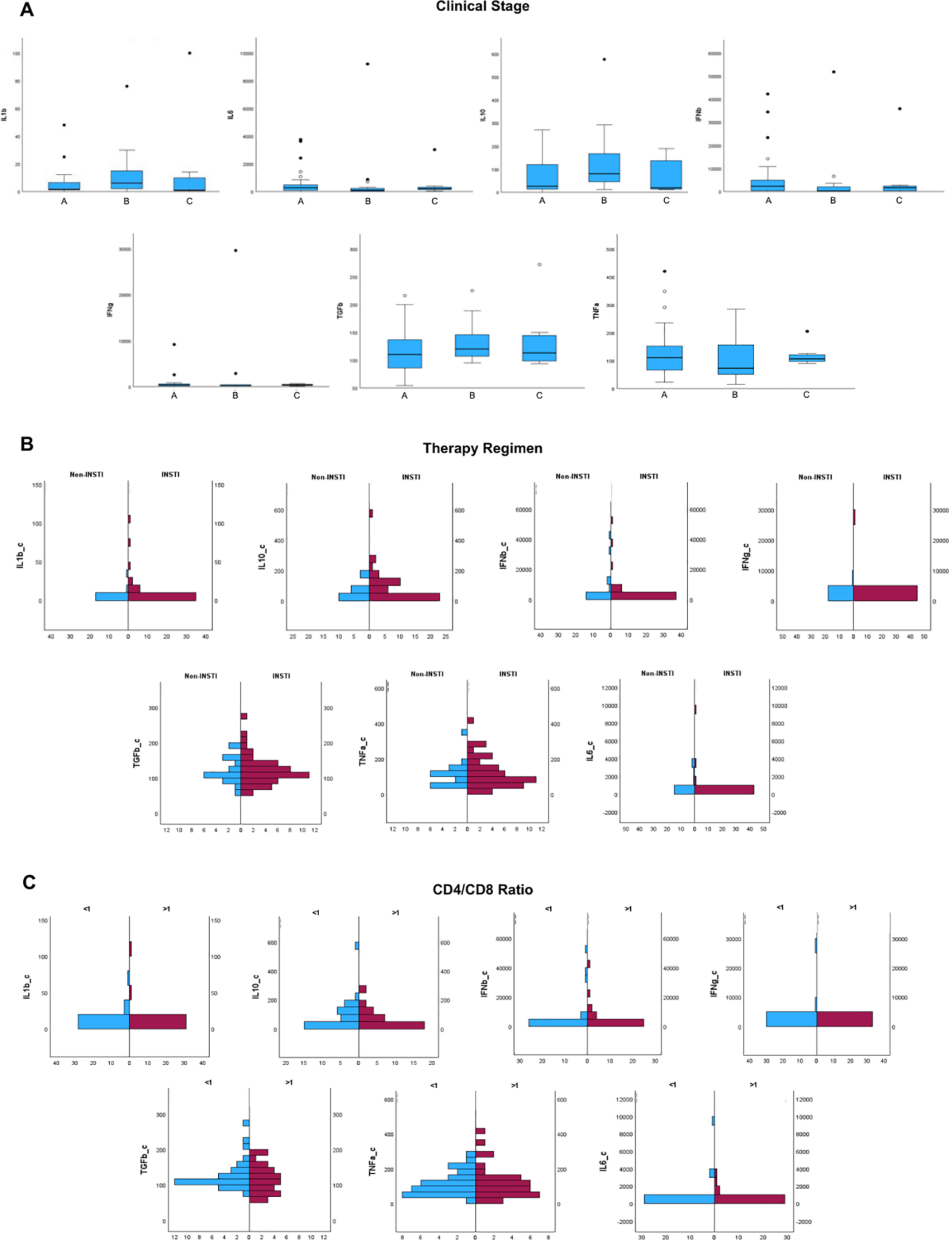
Basal gene expression of immune activation markers in circulating PBMCs. Comparative analysis of pro- and anti-inflammatory cytokine transcripts across participant groups stratified by **(A)** clinical stage, **(B)** antiretroviral regimen (integrase inhibitor–based vs. non–integrase inhibitor–based therapy), and **(C)** CD4/CD8 ratio category.

We subsequently assessed the basal gene expression profile in PBMCs from the same cohort. A targeted panel of genes involved in peripheral immune activation secondary to microbial translocation was analysed to explore systemic correlates of mucosal barrier dysfunction. No significant differences were observed between groups in the expression levels of key pro-inflammatory and anti-inflammatory cytokines, indicating comparable transcriptional activity of major immune mediators across the clinical and therapeutic strata examined (**Figure 3**).

### 3.5 Measurements of enhanced intestinal permeability and systemic inflammation biomarkers in plasma

**Figure 4:**
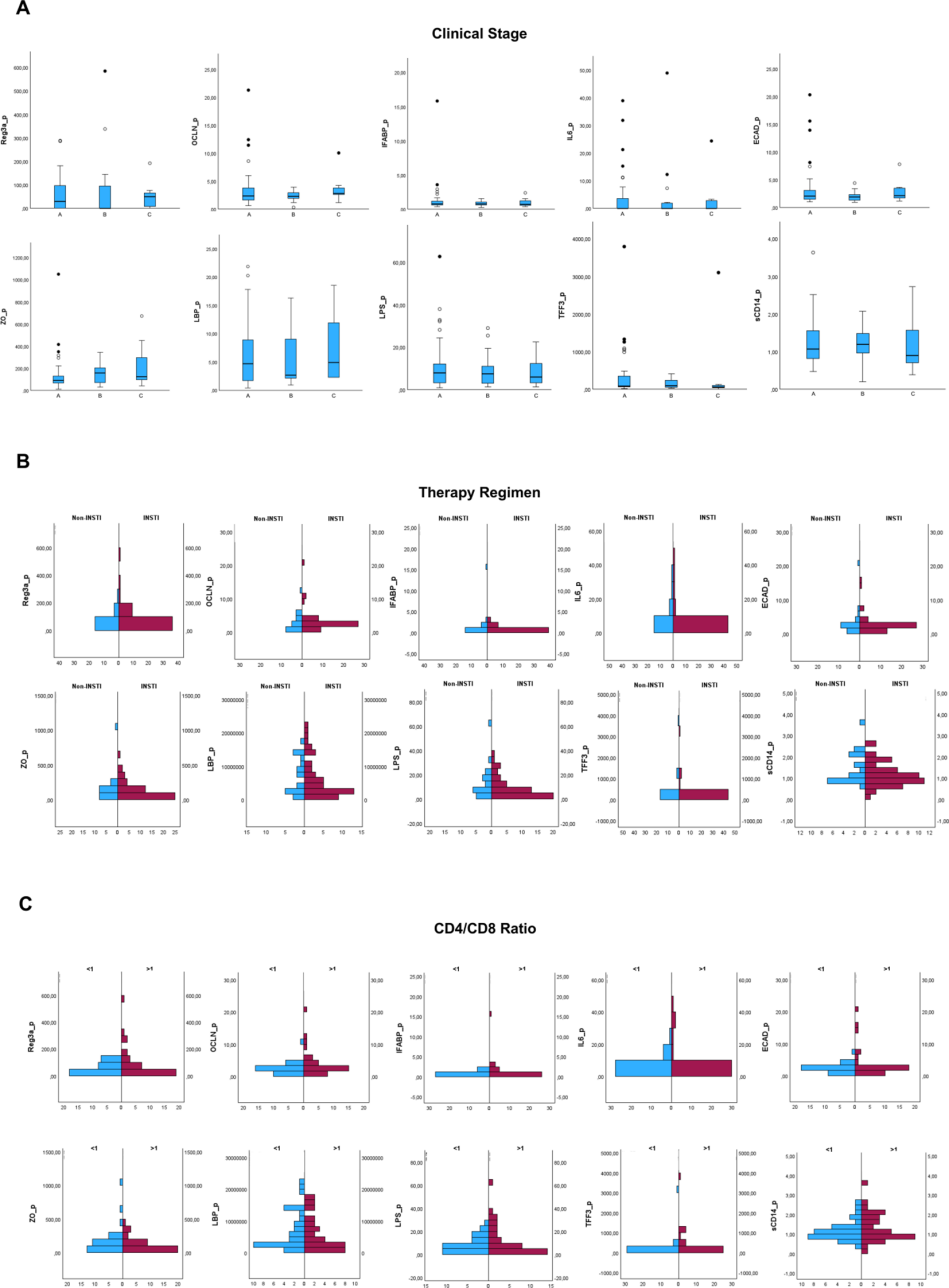
Plasma levels of biomarkers of intestinal epithelial barrier integrity and systemic inflammation across study groups. Comparative analysis of ten circulating analytes measured in participants stratified by **(A)** clinical stage, **(B)** antiretroviral regimen (integrase inhibitor–based vs. non–integrase inhibitor–based therapy), and **(C)** CD4/CD8 ratio category.

We further quantified a panel of circulating biomarkers reflecting intestinal epithelial barrier damage and systemic inflammation in plasma samples from all participants. Absolute concentrations of the ten selected biomarkers were measured and are reported in **Supplementary Figures S3**, and **S4**, which provide detailed quantitative data at the individual-sample level. Statistical analysis did not reveal any significant differences in the levels of these biomarkers among the examined strata. Although certain analytes appeared elevated in individual cases, their overall distribution was heterogeneous and did not display a consistent pattern of group-specific variation (**Figure 4**).

### 3.6 Regression analysis

Linear regression analyses were performed to assess whether differences in mucosal gene expression persisted after adjustment for relevant clinical covariates. In CDC stage analyses, adjusted for CD4^+^ nadir and HIV RNA zenith, *CLDN3* (B = -0.785, SE = 0.279, *p* = 0.007) and *CLDN7* (B = -0.329, SE = 0.167, *p* = 0.05) were independently associated with disease stage (**Supplementary Table S5**). In CD4/CD8 ratio analyses, adjusted for age and CD4^+^ nadir, *SLPI* retained statistical significance (B = 0.600, SE = 0.301, *p* = 0.05) (**Supplementary Table S6**).

### 3.7 Correlation analysis

**Figure 5:**
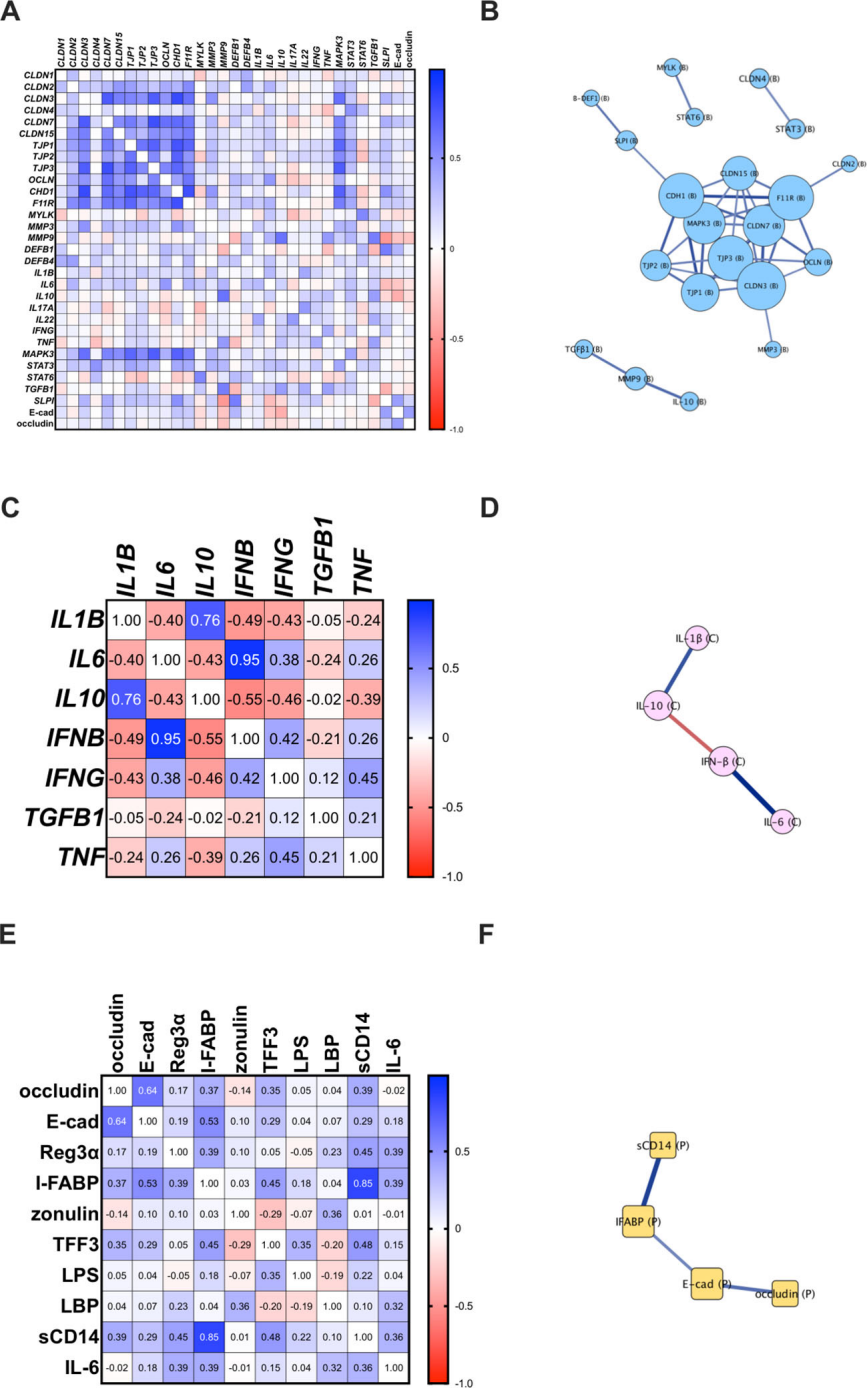
Correlation networks integrating mucosal, peripheral, and systemic parameters. Correlation matrices illustrating pairwise associations among **(A)** gene and protein expression levels in anorectal mucosal biopsies, **(C)** gene expression profiles in PBMCs, and **(E)** plasma biomarkers related to epithelial barrier integrity, microbial translocation, and systemic inflammation. The colour scale indicates the direction of the correlation, with blue denoting positive and red denoting negative associations. **(B, D, F)** show network representations of statistically significant correlations (*p* < 0.05; |r| > 0.5) derived from the matrices presented in **(A, C, E)**, respectively. Each node represents an individual gene, protein, or analyte, and edges denote significant associations. Node size is proportional to the number of connections (degree centrality), highlighting the relative interconnectedness within each network.

We next examined the interrelationships between the expression levels of epithelial barrier–related and immune-related transcripts, including 15 epithelial structural genes, 14 immune-associated genes, and the gene expression of *CHD1* and *OCLN* (**Figure 5A**). Significant positive correlation was observed among multiple genes related to epithelial integrity, including *TJP1*, *TJP2*, *TJP3*, *CLDN3*, *CLDN7*, *CLDN15*, *CHD1*, *OCLN*, *F11R*, and *MAPK3*. Additional positive correlations were identified between *CLDN3*–*MMP3*, *F11R*–*CLDN2*, *CHD1*–*SLPI*–*DEFB1*, *MYLK*–*STAT6*, *CLDN4*–*STAT3*, and *TGFB1*–*MMP9*–*IL10* (**Figure 5B**).

We next extended the correlation analysis to the seven genes associated with systemic immune activation whose expression was quantified in PBMCs (**Figure 5C**). Statistically significant correlations were identified among these transcripts, delineating a coherent regulatory network of cytokine interactions. Specifically, *IL1B* expression correlated positively with *IL10*, while *IL10* showed a significant negative correlation with *IFNB*. In turn, *IFNB* expression correlated positively with *IL6*. (**Figure 5D**).

We further explored the associations among the ten plasma biomarkers indicative of intestinal epithelial damage, microbial translocation, and systemic inflammation (**Figure 5E**). Notably, a general pattern emerged in which markers of epithelial barrier disruption showed positive correlations with those associated with microbial translocation and systemic inflammatory activation. Specifically, occludin plasma levels correlated positively with those of E-cadherin, which in turn correlated positively with I-FABP, and I-FABP correlated positively with sCD14 (**Figure 5F**).

To integrate the multiple layers of data generated in this study, we performed a comprehensive correlation analysis among all measured parameters. This approach enabled the exploration of potential associations between mucosal, peripheral, and systemic compartments, providing an integrated overview of epithelial and immune dynamics within the cohort However, when applying the significance thresholds (*p* < 0.05; |r| > 0.5), no significant correlations were observed between the mucosal and peripheral compartments, nor between the molecular parameters analysed and the different participant groups defined by clinical or immunological characteristics (data not shown).

## 4 Discussion

PLWH under long-term suppressive ART continue to exhibit evidence of epithelial–immune dysregulation at multiple anatomical sites, yet the extent to which mucosal barrier integrity and local immune activation remain perturbed in the anorectal compartment is not fully defined. In this study, we conducted an integrated assessment of epithelial junctional architecture, host-defence gene expression, and systemic biomarkers to delineate residual alterations in mucosal barrier function and their relationship with key HIV-related clinical parameters.

In our cohort, comprising up to 68 individuals depending on data availability, we observed that those at advanced clinical disease stage exhibited reduced expression of tight junction and adherens junction transcripts, including *CLDN3*, *CLDN7*, *CLDN15*, and *TJP3*, in anorectal mucosal biopsies. Importantly, regression analyses indicated that the associations of *CLDN3* and *CLDN7* with clinical disease stage were independent of both CD4^+^ nadir and HIV RNA zenith, suggesting that these epithelial alterations are not solely attributable to markers of HIV disease severity. This observation echoes prior evidence that HIV infection impairs the gut epithelial barrier: in particular, transcript reductions in TJ components have been reported in chronically treated PLWH, suggesting persistent epithelial dysregulation despite viral suppression (29–31). Our data extend this by showing that antiviral drug class (i.e., integrase inhibitor-based regimens) and immune reconstitution status (CD4/CD8 ratio >1) are associated with distinct mucosal gene expression signatures: we found that *CLDN2* down-regulation and *CLDN4* up-regulation occurred in INSTI-treated participants, along with higher expression of *CHD1*, *TJP1*, *TJP3* and innate defence genes (*BDEF1* and *SLPI*) in the CD4/CD8 >1 group. Among these transcripts, only *SLPI* maintained an independent association with CD4/CD8 ratio after adjustment for age and CD4^+^ nadir, highlighting a potential link between mucosal innate defence pathways and the degree of immune reconstitution achieved under suppressive ART. While mechanistic studies remain limited, this suggests that certain ART regimens may exert beneficial effects on barrier gene expression, and that immune recovery (as indicated by CD4/CD8 ratio) aligns with preservation of epithelial transcriptomic architecture. Importantly, in the absence of an HIV-uninfected comparator group, these observations should not be interpreted as evidence of complete restoration of epithelial barrier integrity. Rather, the identified transcriptional differences likely reflect relative variations within a treated HIV population, highlighting factors associated with a more favourable mucosal profile without establishing the extent to which epithelial homeostasis has normalized compared with uninfected individuals.

In the broader field, the role of epithelial junctional integrity as a driver of microbial translocation and systemic immune activation in HIV has been established: reviews highlight that epithelial cell death, loss of TJ proteins, depletion of gut CD4^+^ Th17 cells and microbial dysbiosis all converge to weaken the mucosal barrier (30) (32, 33). Our correlation analyses in mucosal biopsies yield additional insight: the observed strong positive correlations among multiple structural genes (*TJP1/2/3*, *CLDN3/7/15*, *CHD1*, *OCLN*, *F11R*, *MAPK3*) and additional immune–structural pairings (e.g., *CLDN3*–*MMP3*; *CHD1*–*SLPI*–*DEFB1*) indicate a tightly co-regulated transcriptional network. This supports the concept of dynamic interplay between epithelial junctional maintenance and local innate immune responses at the mucosal interface, a nuance that has been less explored in HIV literature.

Focusing on circulating PBMCs we did not observe between-group differences in baseline expression of key cytokine transcripts by clinical stage, ART class, or CD4/CD8 strata. This lack of stratified difference aligns with multiple prior reports that peripheral immune activation metrics often remain elevated or heterogeneous in treated PLWH and do not necessarily stratify according to ART class or immune recovery subgroup (30, 33). However, our correlation network analysis among the seven immune-activation genes revealed significant associations (e.g., *IL1B*–*IL10* positive; *IL10*–*IFNB* negative; *IFNB*–*IL6* positive), delineating a coherent network of cytokine interplay. This suggests that even without major group differences, transcriptional interconnections among inflammatory mediators remain intact and tightly regulated in the peripheral compartment. It underscores that homeostatic modulation of immune responses persists, even in the context of stable ART-suppression.

To assess whether systemic signatures paralleled the mucosal phenotype, we extended our analysis to plasma biomarkers of epithelial damage and immune activation. We quantified a panel of ten plasma biomarkers reflecting intestinal epithelial injury, microbial translocation, and systemic inflammation and found no significant stratification by participant group. This observation mirrors literature showing that in cART-treated cohorts, biomarkers of gut damage and microbial translocation (e.g., I-FABP, REG3α, sCD14, BDG) may have limited discriminatory power across clinical sub-groups (30, 33). It is also conceivable that compensatory mechanisms operating during long-term suppressive ART partially buffer the systemic consequences of ongoing epithelial perturbations, resulting in biomarker levels that remain relatively stable despite underlying mucosal heterogeneity. This interpretation is consistent with the notion that tissue-level dysfunction may persist after systemic inflammatory parameters have reached a new equilibrium under effective treatment. Notwithstanding, our correlation analyses uncovered a positive association continuum: plasma levels of occludin correlated positively with E-cadherin, which in turn correlated with I-FABP, and further with sCD14. This pattern suggests that even in treated PLWH, variations in soluble junctional proteins, enterocyte injury markers and monocyte activation markers remain interconnected, supporting the model whereby barrier disruption, microbial product leakage and immune activation remain functionally linked. This aligns with mechanistic conceptualizations in which epithelial junction compromise is upstream of microbial translocation and downstream immune activation (31) (34).

Interestingly, when applying stringent thresholds (*p* < 0.05 and |r| > 0.5), we did not detect significant correlations between mucosal and peripheral compartments or between molecular parameters and participant stratification groups. This finding reinforces recent views that, in virologically suppressed individuals, mucosal, peripheral and systemic biomarker compartments may operate in partially decoupled fashion, i.e., structural perturbation at the mucosa does not necessarily translate linearly into peripheral transcript change or plasma marker shifts. One possible contributor to this compartmentalization is the persistence of local tissue-specific drivers that are not adequately reflected in the circulation. Among these, residual HIV reservoirs within mucosal tissues have been proposed as potential sources of chronic immune stimulation despite systemic viral suppression, although such parameters were not evaluated in the present study. This may help explain why participants with advanced clinical history or lower CD4/CD8 ratios exhibited detectable mucosal alterations without a correspondingly heightened systemic inflammatory profile. Such findings suggest that, in the setting of durable viral suppression, local epithelial and immune disturbances may persist independently of overt systemic immune activation. An additional consideration is that prolonged virological suppression may attenuate systemic inflammatory signals to a greater extent than tissue-specific perturbations. Under these conditions, residual mucosal abnormalities may persist without necessarily reaching the threshold required to generate measurable differences in circulating immune markers or PBMC transcriptional profiles, thereby contributing to the apparent dissociation between local barrier dysfunction and systemic inflammation observed in our cohort. Studies using *in vivo* permeability imaging have similarly shown lack of robust group-stratified differences in treated individuals (30). Two major implications arise: (1) biomarkers and transcriptomic signatures originating from distinct biological compartments may capture divergent and temporally uncoupled processes, and (2) therapeutic strategies aimed at restoring barrier integrity may require direct targeting of mucosal sites rather than relying exclusively on systemic or circulating biomarkers.

In interpreting these results, the limitations inherent to our study design must also be recognized: its cross-sectional design prevents causal inferences, and the relatively small sample size may limit power to detect subtle between-group differences. This limitation is particularly relevant for the CDC-based analyses, as only six individuals were classified as stage C. Consequently, findings related to disease-stage stratification should be interpreted cautiously and require validation in larger cohorts with a more balanced distribution across clinical categories. In addition, our cohort comprised only virologically suppressed adults and lacks HIV-uninfected controls. Furthermore, although all participants exhibited sustained plasma viral suppression, we did not assess tissue-associated HIV DNA or RNA within mucosal biopsies. Therefore, the potential contribution of local viral persistence to the epithelial and immune signatures observed cannot be excluded. Given the recognized role of tissue HIV reservoirs in maintaining residual immune activation despite suppressive ART, future studies incorporating direct measures of mucosal HIV persistence will be important to determine the extent to which local viral activity contributes to barrier dysfunction and immune dysregulation. Future work should incorporate longitudinal sampling to assess temporal shifts in mucosal, peripheral and plasma markers, and to determine how treatment duration, cumulative viral suppression, and evolving immune recovery influence epithelial–immune dynamics over time. Such approaches will be essential to establish whether the signatures identified here represent persistent abnormalities or progressive restoration trajectories under long-term ART. Lastly, the detailed correlations we describe warrant mechanistic validation *in vitro* and *in vivo* to determine whether the identified transcript networks are functionally causal.

## 5 Conclusions

In summary, our data provide evidence that in PLWH on stable suppressive ART, epithelial junctional gene expression in anorectal mucosa remains responsive to clinical stage, ART class and immune recovery status, while peripheral and circulating marker read-outs are relatively homogeneous across groups. The persistence of coherent transcriptional and soluble biomarker networks within compartments, despite lack of cross-compartment coherence, highlights the complexity of mucosal-immune system interactions in HIV. These findings support the rationale for focusing on mucosal barrier restoration as a potential adjunctive therapeutic strategy in HIV and encourage the integration of compartment-specific read-outs when evaluating gut-targeted interventions.

## Data Availability

The datasets generated and analyzed during the current study are available through the Zenodo repository (DOI: 10.5281/zenodo.20596281). Access to the dataset is restricted and may be granted upon reasonable request to the corresponding authors.
