# Supplementary material for: Mucosal and systemic immune signatures reveal compartmentalized regulation of gut barrier integrity in virologically suppressed HIV Infection
Source: Front Immunol. 2026 Jul 15;17:1876176. doi: 10.3389/fimmu.2026.1876176 (PMC13415938; doi:10.3389/fimmu.2026.1876176)
Supplement: Supplementary file 1 [file Supplementaryfile1.pdf]

Mucosal and Systemic Immune Signatures Reveal Compartmentalized Regulation of Gut Barrier Integrity in Virologically Suppressed HIV Infection

Valentina Artusa[1]✉, Roberta Zamarato[1], Lara De Luca[1], Livia Benedetti[2], Mirko Compagno[2], Mario Alberto Cano Fiestas[3], Diego Bottan[1], Mario Clerici[4,5], Mara Biasin[1] and Daria Trabattoni[1]✉; MARISA Study Group

[1]*Department of Biomedical and Clinical Sciences, University of Milan, Via Giovanni Battista Grassi 74, 20157 Milan, Italy*
[2]*Department of System Medicine, Tor Vergata University, Rome, Italy*
[3]*Department of Clinical Sciences and Community Health, University of Milan, Via della Commenda 19, 20122 Milan, Italy*
[4]*Department of Pathophysiology and Transplantation, University of Milan, Via Francesco Sforza 12, 20122 Milan, Italy*
[5]*IRCCS Fondazione Don Carlo Gnocchi ONLUS, Via Capecelatro 66, 20148 Milan, Italy*
✉ Correspondence to valentina.artusa@unimi.it, daria.trabattoni@unimi.it

Supplementary Material

1.1. Gene Expression Analysis via Real Time-PCR

For each sample, 25 ng of cDNA were used as input for real-time PCR analysis. Gene expression profiling was performed for a total of 32 target genes, as detailed in **Supplementary Table S.1**.

**Housekeeping Genes**	
GAPDH	qHsaCID0015464
β-actin	qHsaCED0036269
RPLP0	qHsaCED0038653
RPS9	qHsaCID0038162
**Structural Genes**	
CLDN1	qHsaCID0006097
CLDN2	qHsaCED0004413
CLDN3	qHsaCED0019605
CLDN4	qHsaCED0038265
CLDN7	qHsaCED0047491
CLDN15	qHsaCED0036397
TJP1 (ZO-1)	qHsaCID0018062
TJP2 (ZO-2)	qHsaCID0006349
TJP3 (ZO-3)	qHsaCID0016898
OCLN	qHsaCED0038290
CDH1	qHsaCID0015365
F11R (JAM-A)	qHsaCED0045829
MYLK (MLCK)	qHsaCED0036325

MMP3	qHsaCID0006170
MMP9	qHsaCID0011597
**Immunological Genes**	
DEFB1	qHsaCID0015106
DEFB4	5’-AGAGTGGAGCCATATGTCATCC-3’ (Forward)<br>5’-TCGCACGTCTCTGATGAGGGAGCCC-3’ (Reverse)
IL1B	qHsaCID0022272
IL6	qHsaCED0044677
IL10	qHsaCED0044704
IL17A	qHsaCID0015941
IL22	qHsaCID0022987
IFNG	qHsaCED0043378
TNF	qHsaCED0037461
MAPK3 (ERK1)	qHsaCID0010939
STAT3	qHsaCID0010912
STAT6	qHsaCED0056844
TGFB1	qHsaCID0017026
SLPI	qHsaCED0044010

Table S.1. Details of the primer pairs used for gene expression analysis. Primer identity is reported either as the reference number of the BIO-RAD PrimePCR™ Assay or, when custom-designed, as the corresponding forward and reverse oligonucleotide sequences.

1.2. Protein purification from RNA STAT-60-homogenized biopsies

Protein isolation was performed from rectal biopsy samples previously homogenized in RNA STAT-60, following an optimized protocol adapted from Wen et al. [1] After the extraction of the RNA-containing upper aqueous phase, residual aqueous content was carefully removed to minimize RNA contamination in the subsequent protein fraction. To precipitate genomic DNA, 180 µL of 100% ethanol was added per 600 µL of RNA STAT-60 to the remaining interphase and organic phase. Samples were vortexed vigorously for 15 seconds, incubated at room temperature for 2–3 minutes, and centrifuged at 5,000 × g for 10 minutes at room temperature. This step resulted in the formation of a gelatinous, transparent DNA pellet.

The protein-containing supernatant was carefully transferred into new sterile microcentrifuge tubes, with a maximum of 450 µL per tube. Tubes containing the DNA pellet were discarded. To precipitate proteins, 650 µL of 100% ethanol was added per 450 µL of protein-rich phase, followed by vortexing. Subsequently, 100 µL of BCP per 450 µL of sample was added and vortexed thoroughly. To facilitate protein precipitation, 600 µL of sterile distilled water was then added per 450 µL of protein phase, and the samples were vortexed again and centrifuged at 12,000 × g for 10 minutes at room temperature.

Following centrifugation, proteins appeared as a whitish, sheet-like precipitate located at the interphase between a pink lower phase and a clear upper aqueous phase. The upper phase was carefully removed without disturbing the protein layer. An additional wash was performed by adding 700 µL of 100% ethanol per 450 µL of original protein phase, followed by vortexing

and centrifugation at 12,000 × g for 5 minutes at room temperature. The resulting protein pellets were briefly air-dried under a laminar flow hood for 2–3 minutes with open tube lids.

Dried pellets were resuspended in SDS-urea complete buffer (85% SDS-urea buffer (#41920158-1, BioPLUS Chemicals), 12% glycerol (G5516, SIGMA Life Science), 2% β-mercaptoethanol (#M7522, SIGMA Life Science), 1% Protease inhibitor cocktail (#P8340, SIGMA Life Science)) to ensure efficient solubilization. To aid complete dissolution, samples were incubated in a thermostatic water bath at 50 °C for 60 minutes. Finally, proteins were denatured by heating at 95 °C for 5 minutes in a thermoblock and subsequently stored at -20 °C until further analysis.

1.3. Distribution analysis of epithelial junctional protein abundance across clinical and immunological stratifications

To further characterize epithelial junctional protein expression at the individual level, we analysed the distribution of densitometric values for the adherens junction protein E-cadherin, the tight junction protein occludin, and their relative ratio across clinical and immunological stratifications. Frequency-based analyses revealed largely overlapping distributions of E-cadherin and occludin protein abundance across groups stratified by clinical disease stage, antiretroviral therapy regimen, and CD4/CD8 ratio, with no significant differences detected at the level of individual proteins.

In contrast, the E-cadherin/occludin ratio was significantly higher in individuals receiving integrase strand transfer inhibitor (INSTI)–based therapy compared with those on non-INSTI regimens, indicating a relative shift toward preservation of adherens junction components within this treatment group. No significant differences in the E-cadherin/occludin ratio were observed when stratifying by clinical disease stage or CD4/CD8 ratio. Together, these findings suggest that ART class may selectively influence the balance of epithelial junctional components without markedly altering absolute protein abundance.

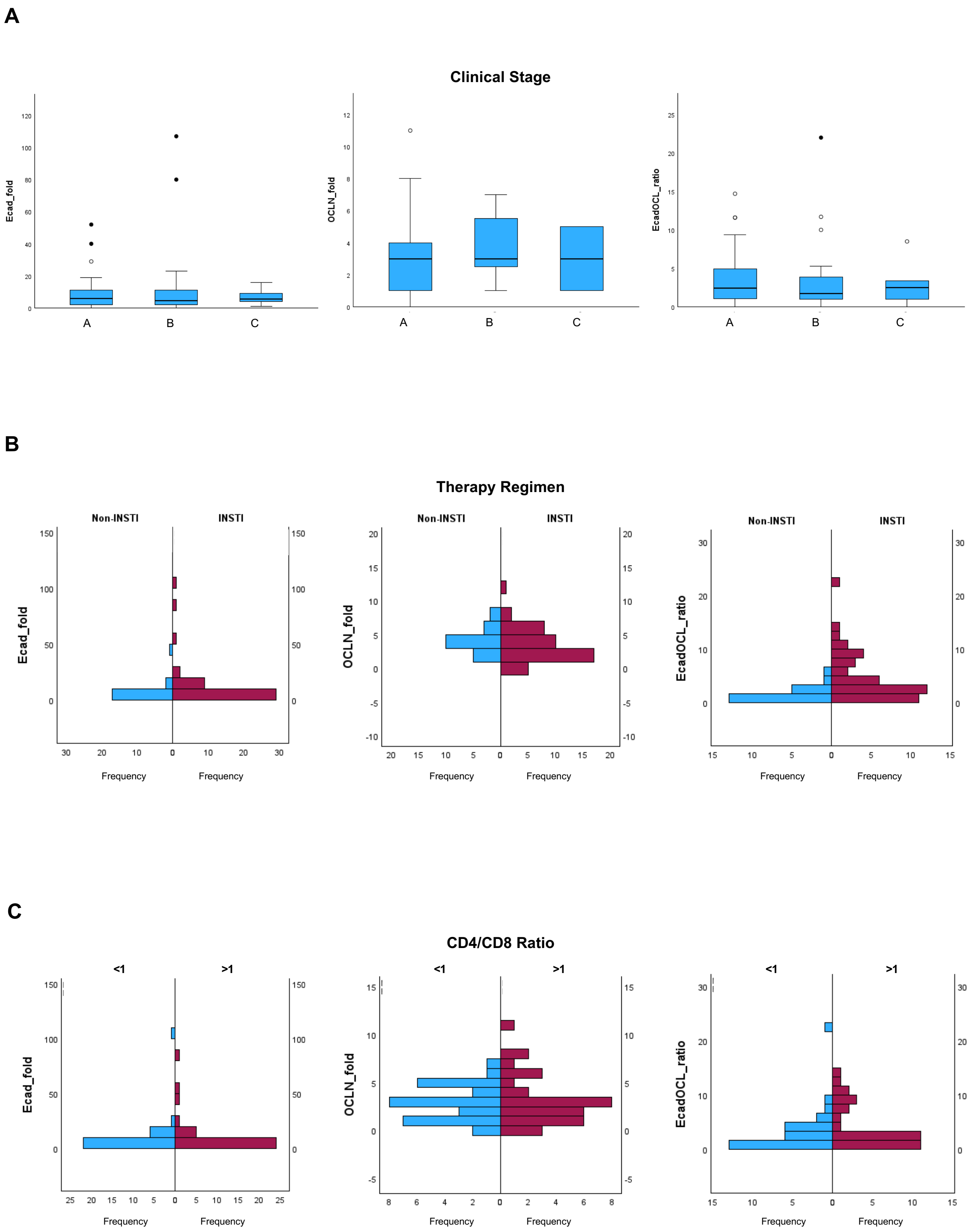

Figure S.2. Distribution of epithelial junctional protein abundance and E-cadherin/occludin ratio across clinical and immunological stratifications. Frequency plots showing the distribution of densitometric values for E-cadherin, occludin, and the E-cadherin/occludin ratio in anorectal mucosal biopsies. Data are displayed according to stratification by clinical disease stage, antiretroviral therapy regimen (INSTI-based vs. non-INSTI), and CD4/CD8 ratio groups. Densitometric values were obtained from Western blot analyses and normalized to the appropriate loading control. While individual E-cadherin and occludin protein levels show overlapping distributions across all stratifications, the E-cadherin/occludin ratio is significantly increased in the INSTI-based therapy group.

1.4. Absolute plasma concentrations of intestinal permeability and systemic inflammation markers

In addition to the group-based comparisons presented in the main manuscript, the absolute plasma concentrations of all ELISA-measured analytes for each individual sample are reported. Specifically, individual-level concentrations of the six markers related to intestinal epithelial integrity and permeability (Occludin, E-cadherin, I-FABP, REG3α, TFF3, and Zonulin,) are shown in **Figure S.3**, while concentrations of the four markers reflecting microbial translocation and systemic inflammation (LPS, LBP, sCD14, and IL-6) are reported in **Figure S.4**. These data provide a detailed overview of the inter-individual variability within the study cohort and complement the comparative analyses presented in the main text.

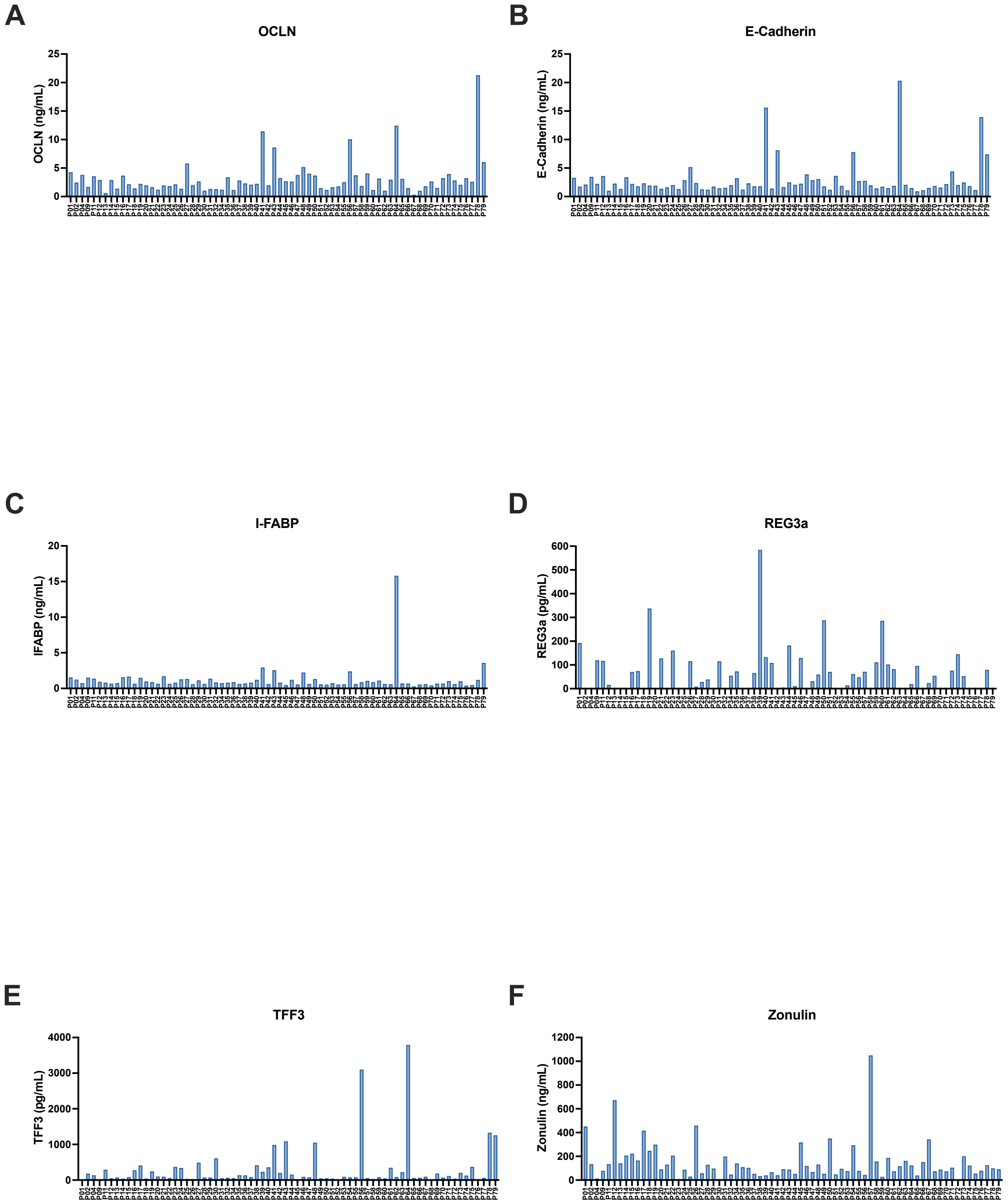

Figure S.3. Plasma concentrations of intestinal epithelial integrity and permeability markers. Bar graphs showing individual plasma concentrations of Occludin, E-cadherin, I-FABP, REG3α, TFF3, and Zonulin, measured by ELISA in plasma samples from the study cohort. Each bar represents one individual sample. Absolute concentrations were calculated based on manufacturer-provided standard curves and illustrate inter-individual variability across the cohort.

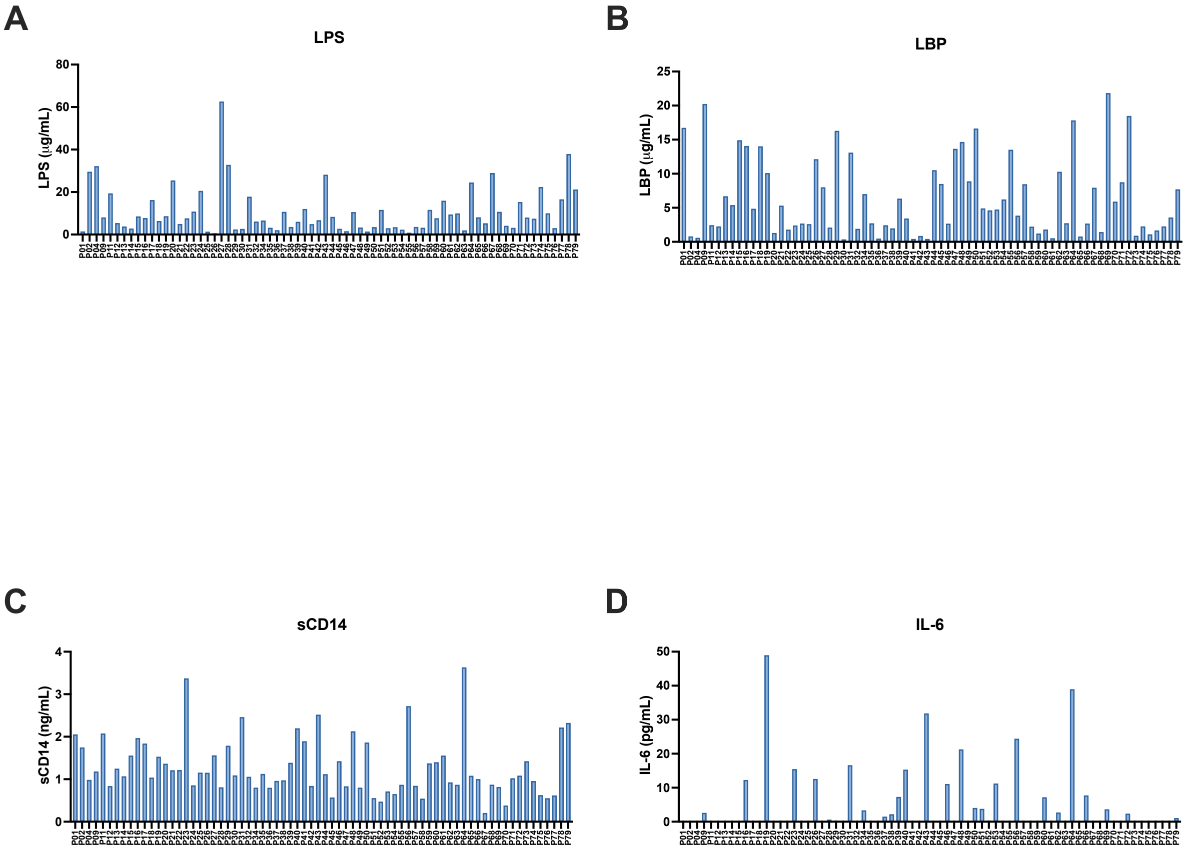

Figure S.4. Plasma concentrations of markers of microbial translocation and systemic inflammation. Bar graphs showing individual plasma concentrations of LPS, LBP, soluble CD14 (sCD14), and IL-6 measured by ELISA in plasma samples from the study cohort. Each bar represents one individual sample. Absolute concentrations are reported to reflect variability in microbial translocation and systemic inflammatory status within the cohort.

1.5. Regression analysis coefficients

Coefficient values of regression analysis (described in Manuscript paragraph 3.6) are listed in **Tables S.5** and **S.6**.

**Variable**	***B***	**SE (*B*)**	***β***	**95% CI**		***t***	***p***
CLDN3	-0,785	0,279	-0,433	-1,343	-0,226	-2,816	**0,01**
CLDN1	1,501	0,522	0,718	0,404	2,598	2,876	**0,01**
MYLK	-0,672	0,255	-0,414	-1,184	-0,160	-2,630	**0,01**
IFNg	0,657	0,262	0,428	0,129	1,184	2,508	**0,02**
MAPK3	-0,361	0,149	-0,388	-0,660	-0,062	-2,421	**0,02**
CLDN7	-0,329	0,167	-0,304	-0,663	0,004	-1,977	**0,05**
CLDN15	-0,402	0,211	-0,305	-0,826	0,021	-1,904	0,06
TGFB1	-0,355	0,205	-0,278	-0,766	0,057	-1,730	0,09
TJP3	-0,328	0,198	-0,272	-0,725	0,069	-1,659	0,10
IL17A	0,888	0,531	0,610	-0,219	1,996	1,673	0,11
IL22	0,590	0,367	0,422	-0,165	1,345	1,606	0,12
IL1b	0,227	0,163	0,210	-0,101	0,555	1,388	0,17

IL6	0,443	0,321	0,229	-0,202	1,087	1,377	0,17
STAT6	-0,161	0,136	-0,198	-0,435	0,112	-1,185	0,24
MMP3	-0,446	0,440	-0,208	-1,338	0,446	-1,014	0,32
CLDN2	-0,325	0,365	-0,177	-1,063	0,412	-0,890	0,38
BDEF2	0,414	0,485	0,169	-0,563	1,391	0,854	0,40
TNFa	0,253	0,308	0,147	-0,365	0,870	0,822	0,42
MMP9	-0,247	0,305	-0,137	-0,859	0,365	-0,809	0,42
CLDN4	0,497	0,778	0,204	-1,143	2,138	0,640	0,53
ECAD	-0,132	0,213	-0,100	-0,559	0,294	-0,622	0,54
SLPI	-0,152	0,261	-0,096	-0,675	0,371	-0,582	0,56
TJP1	-0,105	0,182	-0,097	-0,469	0,259	-0,579	0,57
BDEF1	0,174	0,362	0,083	-0,552	0,900	0,482	0,63
F11R	0,049	0,159	0,050	-0,271	0,368	0,306	0,76
TJP2	-0,034	0,180	-0,031	-0,395	0,328	-0,187	0,85
IL10	-0,030	0,256	-0,023	-0,546	0,485	-0,119	0,91
OCLN	0,022	0,220	0,016	-0,420	0,464	0,099	0,92
STAT3	-0,001	0,114	-0,002	-0,229	0,227	-0,009	0,99

Table S.5. Multivariable linear regression analysis of clinical stage stratification and mucosal gene expression. Regression models were adjusted for HIV RNA zenith and CD4+ nadir. Data are presented as unstandardized regression coefficients (*B*) with their corresponding standard errors (SE (*B*)), standardized regression coefficients (*β*), and 95% confidence intervals (95% CI). The *t*-statistic (*t*) represents the Student's *t*-test value for the predictor, and the *p*-value (*p*) indicates the two-sided statistical significance of the association, evaluated at an alpha level of 0.05. Dependent variables were natural log-transformed prior to analysis.

**Variable**	***B***	**SE (*B*)**	***β***	**95% CI**		***t***	***p***
SLPI	0,6	0,301	0,264	-0,003	1,202	1,995	**0,05**
ECAD	0,475	0,25	0,253	-0,027	0,976	1,897	0,06
TJP1	0,364	0,209	0,233	-0,054	0,782	1,745	0,09
BDEF1	0,732	0,421	0,24	-0,112	1,577	1,739	0,09
CLDN7	0,32	0,21	0,196	-0,101	0,741	1,523	0,13
TJP2	0,3	0,21	0,195	-0,12	0,72	1,433	0,16
CLDN3	0,446	0,345	0,168	-0,245	1,137	1,292	0,20
MAPK3	0,228	0,185	0,163	-0,142	0,599	1,234	0,22
F11R	0,237	0,193	0,164	-0,148	0,623	1,233	0,22
OCLN	0,316	0,264	0,161	-0,213	0,845	1,198	0,24
IL6	-0,453	0,386	-0,161	-1,227	0,32	-1,174	0,25
TJP3	0,264	0,233	0,156	-0,204	0,732	1,131	0,26
IL1b	-0,241	0,215	-0,154	-0,673	0,191	-1,117	0,27
MMP9	-0,311	0,367	-0,117	-1,046	0,424	-0,848	0,40
CLDN15	0,204	0,253	0,109	-0,302	0,711	0,809	0,42
TNFa	-0,265	0,352	-0,108	-0,97	0,441	-0,753	0,46
MMP3	-0,393	0,539	-0,124	-1,483	0,698	-0,729	0,47
IL22	-0,298	0,424	-0,133	-1,169	0,573	-0,703	0,49

BDEF2	-0,317	0,594	-0,083	-1,514	0,881	-0,533	0,60
CLDN4	-0,443	0,888	-0,109	-2,308	1,421	-0,5	0,62
STAT6	0,071	0,162	0,061	-0,254	0,396	0,437	0,66
CLDN2	-0,153	0,425	-0,054	-1,009	0,703	-0,361	0,72
MYLK	0,105	0,324	0,045	-0,544	0,754	0,324	0,75
IL17A	-0,171	0,615	-0,067	-1,454	1,112	-0,278	0,78
IFNg	-0,064	0,342	-0,029	-0,752	0,625	-0,186	0,85
CLDN1	0,149	0,843	0,048	-1,616	1,914	0,177	0,86
STAT3	-0,023	0,134	-0,024	-0,293	0,246	-0,173	0,86
IL10	-0,031	0,281	-0,017	-0,596	0,534	-0,111	0,91
TGFB1	0,012	0,252	0,007	-0,493	0,518	0,049	0,96

Table S.6. Multivariable linear regression analysis of CD4/CD8 ratio stratification and mucosal gene expression. Regression models were adjusted for age and CD4+ nadir. Data are presented as unstandardized regression coefficients (*B*) with their corresponding standard errors (SE (*B*)), standardized regression coefficients (*β*), and 95% confidence intervals (95% CI). The *t*-statistic (*t*) represents the Student's *t*-test value for the predictor, and the *p*-value (*p*) indicates the two-sided statistical significance of the association, evaluated at an alpha level of 0.05. Dependent variables were natural log-transformed prior to analysis.

References

[1] Y. Wen, I. J. Vechetti Jr, T. R. Valentino, and J. J. McCarthy, "High-yield skeletal muscle protein recovery from TRIzol after RNA and DNA extraction," *Biotechniques*, vol. 69, no. 4, pp. 264–269, Oct. 2020.
